# Control Method of the Dual-Winding Motor for Online High-Frequency Resistance Measurement in Fuel Cell Vehicle

**DOI:** 10.3390/s22052051

**Published:** 2022-03-06

**Authors:** Cheng Chang, Yafu Zhou, Jing Lian, Jicai Liang

**Affiliations:** State Key Laboratory of Structural Analysis for Industrial Equipment, Faculty of Vehicle Engineering and Mechanics, School of Automotive Engineering, Dalian University of Technology, Dalian 116024, China; dlutcc@mail.dlut.edu.cn (C.C.); lianjing@dlut.edu.cn (J.L.); liangjicai@126.com (J.L.)

**Keywords:** dual-winding motor, high-frequency resistance measurement, input current control, fuel cell vehicle

## Abstract

The dual-winding motor drive has recently been proposed in the field of fuel cell vehicles due to its performance and high robust advantages. Efforts for this new topology have been made by many researchers. However, the high-frequency resistance measurement of a proton exchange membrane fuel cell based on dual-winding motor drive architecture, which is important for water management to optimize the lifespan of fuel cells, was not employed in earlier works. In this paper, a new control method of the dual-winding motor is proposed by introducing a dc input current control to realize high-frequency resistance measurement and normal drive control simultaneously, without using extra dc-dc converter. On the basis of the revealed energy exchange principles among electrical ports and mechanical port of the dual-winding motor, the load ripple caused by high-frequency current perturbation is optimized based on the *q*-axis current distribution between two winding sets. The decoupling control algorithm for the coupling effect within and across windings is also discussed to improve the dynamic response during high-frequency resistance measurement. Finally, simulation results verify the effectiveness and improvement of the proposed method. Fast Fourier transform results indicated that the total harmonic distortion of the dc input current was reduced from 22.53% to 4.47% of the fundamental, and the torque ripple was suppressed from about ±4.5 Nm to ±0.5 Nm at the given operation points.

## 1. Introduction

### 1.1. Water Management of PEMFC

The proton exchange membrane fuel cell (PEMFC) is a promising energy convert device for automotive applications due to its low operation temperature, quick start up, and high-power density [1]. After continuous optimization, significant progress for PEMFC has been made. However, durability and cost are still two main obstacles to realize global commercialization [2]. Since the electrochemical reaction of the fuel cell is carried out at the water–gas–proton three-phase interface, maintaining water balance inside the membrane is important for PEMFC’s durability because excessive or insufficient water in the membrane may lead to a flooding or drying phenomena of the electrolyte membrane [3]. Flooding causes transport obstruction for reactant gases to the catalyst, and drying decreases the membrane ionic conductivity. The hydrogen or air stream must be properly humidified during the operation according to the water content in the fuel cell. Therefore, water management of PEMFC is one of the key factors to improve lifespan. 

### 1.2. Measurement for the Internal Resistance of PEMFC 

However, the inside water content is difficult to be directly measured. Former efforts have been made to estimate the water content indirectly, and revealed that the internal resistance of the membrane is a good indicator of the humidification state [4]. So far, online measurement of the internal resistance has become an important research direction for water management of PEMFC. Among the various measurement techniques, such as electrochemical impedance spectroscopy (EIS), current interrupt, polarization curve, current sweep method and so on, EIS has been widely used due to its accuracy in identifying individual contributions to the total ac impedance from different electrode processes [5]. The principle of EIS is to inject a small amplitude sinusoidal current/voltage perturbation in a broad range of frequencies, measure the resultant voltage/current variation, and then calculate the ac impedance by numerical algorithm. However, EIS technique is time-consuming, expensive, and normally used as a laboratory tool for offline diagnosis. Whereas high-frequency resistance (HFR) measurement [6], a simplified variant of EIS, is only needed to inject a small amplitude sinusoidal high-frequency perturbation in a certain frequency other than a broad frequency range, and HFR is then calculated as the real component of the ac impedance. As a result, HFR measurement is preferred in PEMFC’s online diagnosis and has been well investigated by former researchers.

### 1.3. Conventional Method of HFR Measurement Based on DC/DC Converter

**Figure 1:**
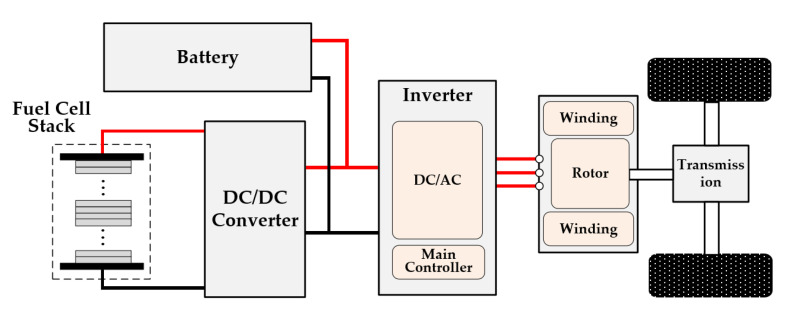
Schematic of HFR measurement based on the dc-dc converter in the conventional drive system.

For the conventional drive system of a fuel cell vehicle, the dc-dc converter was adopted between the PEMFC and the load, acting as a power distributor and voltage adapter [7]. It provided a favorable functionality integration solution into the dc-dc converter for HFR measurement by adding perturbation signals into the normal power conversion process, as shown in Figure 1. The shaping of the PEMFC current can be accurately controlled by the dc-dc converter through regulating its output voltage.

Bethoux et al. [8] presented an imbricate control method to realize the voltage regulation and EIS perturbation simultaneously by a one-freedom PWM control of the dc-dc converter, and found that the injection of the perturbation signal into the inner control loop or outer one depends on the desired frequency. Kitamura et al. [9] implemented on-line HFR measurement under 300 Hz and presented the correlation between internal resistance and water content from the experimental result. Besides the above functionality integration solutions, Hong et al. [10] proposed an add-on solution for HFR measurement by paralleling an extra dc-dc converter to PEMFC. In order to improve the signal/noise ratio for extraction of the current perturbation and voltage response at 320 Hz, the signal processing circuit based on high common-mode differentiation was proposed and validated. Compared with functionality integration solutions, add-on solution is simple and independent on the drivetrain topology, but expensive due to the use of an extra dc-dc converter.

### 1.4. New Drive Architecture Based on Dual-Winding Motor without DC-DC Converter

**Figure 2:**
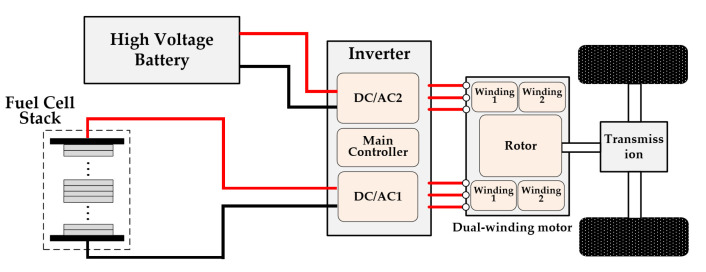
Schematic of the new drivetrain based on the dual-winding motor.

Recently, new drive architectures for fuel cell vehicles are being investigated to meet the increasing needs of performance improvement and cost reduction. Among the different architectures, the dual-winding motor (DWM) drive has attracted attention since it was proposed [11] due to its advantages of lower dc-link current ripple, fault tolerance and better efficiency [12,13]. The dual-winding motor is powered by two inverters with reduced ratings fed by two power sources, a fuel cell stack and a battery pack; thus, the dc-dc converter can be eliminated, as shown in Figure 2. Efforts have been made over the past few years for a dual-winding motor and system integration in fuel cell vehicles, such as a dual-winding motor control method [14], a faulty operation application [13], a cross-coupling cancellation control [15], arbitrary power sharing control [16], energy management strategy [17], and so on. However, the control methods for the HFR measurement of a fuel cell based on this dual-winding motor drive system are not addressed in previous research.

### 1.5. Contributions and Organization

HFR measurement of the PEMFC in a fuel cell vehicle using a DWM drive system architecture is important for water management and meaningful for improving the durability of fuel cells, but it has not been addressed in previous research. Under this background, this paper proposed a new control method for the dual-winding motor to realize HFR measurement and normal drive control simultaneously without using a high-cost dc-dc converter. The main feature was the generation of a high-frequency current perturbation with low total harmonic distortion by introducing an extra input current control loop for the dc link of the PEMFC based on the conventional phase current control method for the motor. However, this will inevitably cause torque ripple due to the fluctuation on the *q*-axis current in the inner phase current control loop. To solve this problem, this paper further provides a control method to optimize the torque ripple based on the investigated energy exchange principles among electrical ports and the mechanical port of the dual-winding motor. In addition, the coupling effect within and across windings was investigated to improve the dynamic response of the inner phase current control loop during HFR measurement. Thus, this paper represents a promising solution of water management for the fuel cell vehicle based on a DWM drive system in a more economical way. 

The rest of this paper is organized as follows. Section 2 introduces the working mode and basic framework of the proposed dual-winding motor drive system. Section 3 analyzes the impedance property of PEMFC by using Randle’s equivalent circuit model. In addition, the model of the dual-winding motor is established considering the coupling effect between two winding sets. In Section 4, the proposed control method is elaborated. In addition, the study reveals important principles for the energy exchange process among electrical ports and the mechanical port. In Section 5, simulation results are discussed and compared. Finally, conclusions of this study are summarized in Section 6.

## 2. System Architecture

**Figure 3:**
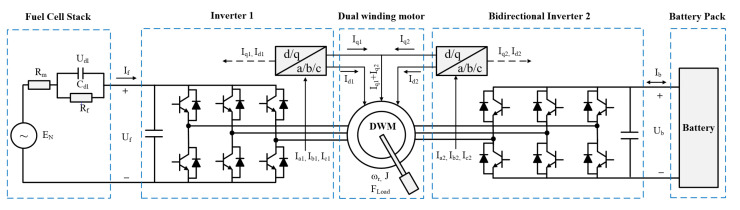
System architecture of the DWM drive system for fuel cell vehicle.

A schematic of the dual-winding motor drive system is illustrated in Figure 3. DWM has two sets of three-phase windings and a shared rotor that constitutes an electromechanical system with three energy ports. Energy flow among the three ports can be regulated by controlling two winding sets synergistically [18]. 

Water distribution inside the membrane of the PEMFC equilibrates slowly during transient states [19]. Sudden changes in the output current can cause a variation of the water and gas content inside the PEMFC, which is the driving force for an extensive lifespan degradation. Therefore, PEMFC shall provide a steady state current as possible during the driving cycle, whereas the battery is mainly responsible for delivering the transient power, acting as an energy buffer. Typically, DWM is operated in an unbalanced way dominated by the power distribution strategy of the vehicle. To avoid diverting the focus of this paper, the power distribution strategy will not be discussed in the paper. As it is fundamental to our research, the system model will be established and elaborated in the following:

The operation modes can be categorized into three types: single-source mode, dual-source mode, and energy transfer mode, as shown in Table 1. In Table 1, O stands for releasing energy and I stands for absorbing energy. In the single-source and dual-source modes, no energy is internally transferred between the two windings sets. While in the energy transfer, modes R1 and R2, the extra power of a PEMFC, will charge the battery when the SOC is low under both traction and braking conditions. 

## 3. Modeling

### 3.1. PEMFC Model

**Figure 4:**
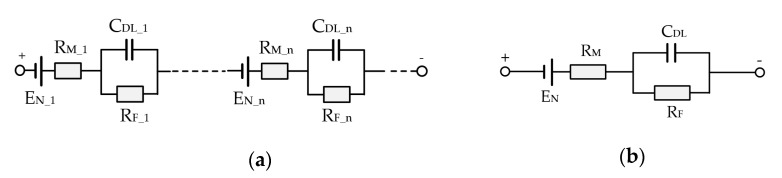
Randle’s equivalent circuit model: (**a**) Presented by individual parameter of each fuel cell; (**b**) presented by consolidated parameter of each fuel cell.

Among different models, the Randle’s equivalent circuit model is commonly applied to describe the electrochemical cells due to its simplicity and physical interpretation of the parameters [20]. As shown in Figure 4a, each fuel cell model is composed of Nernst voltage *E_N_*, double-layer capacitor *C_DL_*, catalyst layer faradic resistance *R_F_*, and membrane ohmic resistance *R_M_*. Neglecting the inconsistencies between cells, the model of the whole fuel cell stack can be consolidated as show in Figure 4b. The parameters with upper case subscripts stand for the consolidated ones for the PEMFC stack.

There must be sufficient water content in the membrane, since the proton conductivity is directly proportional to the water content, whereas too much water will lead to electrolyte flood and blocking of the pores in the electrodes or the gas diffusion layer. Because *R_M_* is strongly correlated with water content, the flooding or drying status of the membrane can be diagnosed by the identification of parameter *R_M_*, which is important for water management to improve the durability of fuel cells.

Understanding the impedance property of this circuit model helps to establish a suitable algorithm for identifying the internal resistance *R_M_*. The transfer function of this circuit can be given by Equation (1). Further, the amplitude and phase angle characteristics in frequency domain can be given by Equations (2) and (3):

(1) $$G\left(s\right)=\frac{U\left(s\right)}{I\left(s\right)}=\left(R_{M}+R_{F}\right)\frac{k_{R}Ts+1}{Ts+1}\;$$

(2) $$\left|G\left(j\omega \right)\right|=\left(R_{M}+R_{F}\right)\sqrt{\frac{1+{\left(\omega k_{R}T\right)}^{2}}{1+{\left(\omega T\right)}^{2}}}\;$$

(3) $$\varphi \left(\omega \right)=\arctan\left(k_{R}T\omega \right)-\arctan\left(T\omega \right)$$

**Figure 5:**
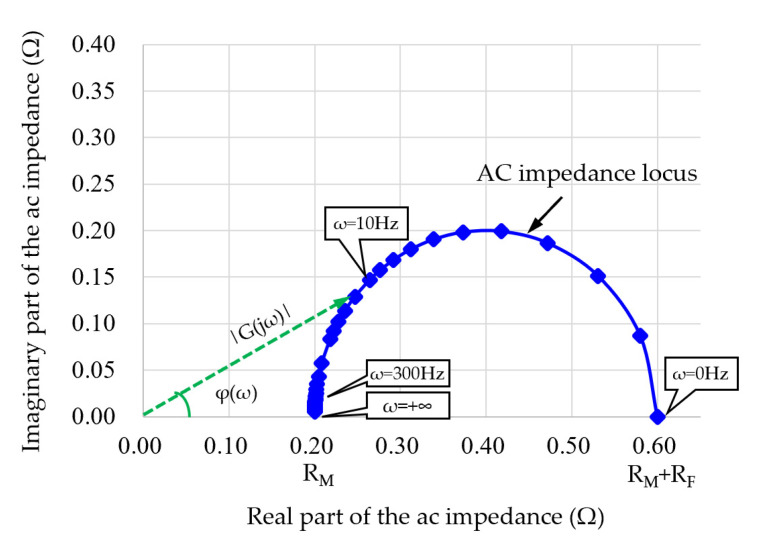
Nyquist plot for the ac impedance of the PEMFC stack under different frequencies.

In the above equations, *ω* is the frequency of the excitation, time constant $T=R_{F}C_{DL}$, and ratio $k_{R}=R_{M}/\left(R_{M}+R_{F}\right)$. The ac impedance property under different frequencies can be expressed by a Nyquist plot, as shown in Figure 5. It can be seen that when the frequency of excitation is high enough, total ac impedance is approximately equal to the internal resistance *R_M_*. This conclusion is important for HFR measurement technology, especially for online applications. Therefore, to achieve the HFR measurement of the fuel cell under dynamic conditions of the vehicle, a high frequency (for example, 300 Hz in this paper) current perturbation shall be imposed on the PEMFC.

### 3.2. Dual-Winding Motor Model

As for the dual-winding motor, the relationship between space vectors of phase voltage $v_{s}^{s}$, flux linkage $\psi_{s}^{s}$, and phase current $i_{s}^{s}$ can be expressed in a UVW stationary reference frame in Equations (4) and (5) under the following assumptions:

- The magnetic saturation and leakage inductance are neglected;
- Two winding sets have the same parameters and no electrical angular displacement in place ($\beta_{1}=\beta_{2}$) to maximize the mutual inductance and energy exchange efficiency;
- Two winding sets are wound on the same iron core with isolated neutral points;
- Current circulation between each phase is eliminated through certain winding design.

(4) $$\{\begin{array}{c} u_{s1}^{s}=R_{1}i_{s1}^{s}+p\left(L_{1}^{s}\cdot i_{s1}^{s}+L_{m}^{s}\cdot i_{s2}^{s}+\omega \Psi_{f}\right)\\ \begin{array}{c} u_{s2}^{s}=R_{2}i_{s2}^{s}+p\left(L_{2}^{s}\cdot i_{s2}^{s}+L_{m}^{s}\cdot i_{s1}^{s}+\omega \Psi_{f}\right) \end{array} \end{array}$$

(5) {ψs1s=L1s·is1s+Lms·is2s+ωΨf ψs2s=L2s·is2s+Lms·is1s+ωΨf

where *R* is the winding resistance, *p* is the time derivative operator, *L* is the self-inductance of each winding, *L_m_* is the mutual inductance between two windings, *ω* is the electric speed, and *Ψ_f_* is the space vector of the permanent magnet flux linkage. Subscripts 1 and 2 represent winding 1 and 2.

Different from the three-phase motor, the dual-winding motor has two separate winding sets fed by two power sources and share the same stator and rotor. However, the *d–q* model of the dual-winding motor can be considered as two *d–q* sets of conventional three-phase motors; thus two separate *d–q* sets are performed as *d*1*–q*1 and *d*2*–q*2 models based on the double *d–q* approach [21]. Based on the double *d–q* approach, the voltage and flux linkage space vector of the stator are then converted into two *d–q* rotating reference frames by using the well-known Clark and Park transformation [22,23]. The phase voltage equations can be transformed from the UVW reference frame into a *d–q* rotational reference frame:

(6) $$u_{d1}=R_{1}i_{d1}+L_{d1}\frac{di_{d1}}{dt}-\omega L_{q1}i_{q1}+L_{md}\frac{di_{d2}}{dt}\;$$

(7) $$u_{q1}=R_{1}i_{q1}+L_{q1}\frac{di_{q1}}{dt}+\omega L_{d1}i_{d1}+\omega \Psi_{f}+L_{mq}\frac{di_{q2}}{dt}\;$$

(8) $$u_{d2}=R_{2}i_{d2}+L_{d2}\frac{di_{d2}}{dt}-\omega L_{q2}i_{q2}+L_{md}\frac{di_{d1}}{dt}$$

(9) $$u_{q2}=R_{2}i_{q2}+L_{q2}\frac{di_{q2}}{dt}+\omega L_{d2}i_{d2}+\omega \Psi_{f}+L_{mq}\frac{di_{q1}}{dt}.$$

The flux linkage equation can be transformed into the *d–q* rotational reference frame:

(10) $$\Psi_{d1}=L_{d1}i_{d1}+L_{md}i_{d2}+\Psi_{f}\;$$

(11) $$\Psi_{q1}=L_{q1}i_{q1}+L_{mq}i_{q2}$$

(12) $$\Psi_{d2}=L_{d2}i_{d2}+L_{md}i_{d1}+\Psi_{f}\;$$

(13) $$\Psi_{q2}=L_{q2}i_{q2}+L_{md}i_{q1}.$$

The above voltage and flux linkage equations revealed that there is a coupling effect between two winding sets resultant from mutual inductance and current transients. Hence, DWM cannot be completely equivalent to two independent motors. Further, the output mechanical power of DWM can be given by:

(14) $$P_{M}=\omega T_{M}=\frac{3}{2}\omega p_{0}\left(\Psi_{d1}i_{q1}-\Psi_{q1}i_{d1}+\Psi_{d2}i_{q2}-\Psi_{q2}i_{d2}\right)$$

where $p_{0}$ are the motor pole pair numbers and *T_M_* represents the output torque. Equation (14) indicates that the power produced by each winding is the product of its current and corresponding flux, which is the same as the traditional three-phase permanent magnet motor. The total electromagnetic torque equals the sum of the torque generated by each winding, and the *d–q*-axis current of the two windings sets can be controlled independently. Therefore, DWM can be equivalent to two three-phase motors in the control aspect.

## 4. Control System and Method

### 4.1. High-Frequency Current Generation by Input Current Control

**Figure 6:**
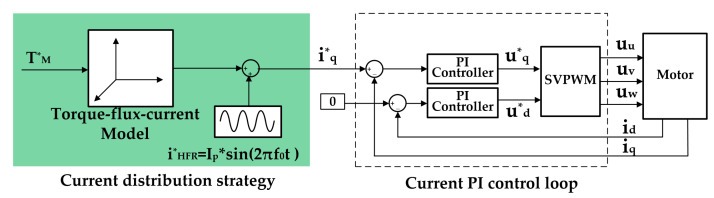
Realization of HFR perturbation current based on the conventional control method.

As aforementioned, DWM can be deemed as two independent three-phase motors in the control aspect. Field-oriented control based on the double *d–q* approach was adopted. To generate HFR perturbation current without using dc-dc converter, one method is to impose an extra high-frequency amount on the motor phase current. The input side of the motor system, that is, the output side of the PEMFC, naturally generates a high-frequency current fluctuation. This is easy to be implemented since the traditional control method takes the motor phase current as the control target based on field-oriented control, as shown in Figure 6. However, this may lead to deterioration of the PEMFC current suffering from the indirect dc current control as well as nonlinearity of the inverter and uncertainty of the system parameters. Deterioration of the PEMFC current influences not only the accuracy of the HFR measurement but also the lifespan of fuel cells.

Considering that a non-salient pole permanent magnet motor was selected in this paper, the *d*-axis current $i_{d1}$ and $i_{d2}$ shall always stay at zero. Therefore, according to Equation (14), the output mechanical power of DWM can be simplified to:

(15) $$P_{M}=\frac{3}{2}\omega p_{0}\Psi_{f}\left(i_{q1}+i_{q2}\right)\;,$$

under the condition that the actual value of the *d*-axis current $i_{d1}$ and $i_{d2}$ are approximately equal to zero. Equation (15) reveals important principles for the energy exchange process among two winding sets (electrical ports) and the vehicle (mechanical port):

- The mechanical power is determined by the overall *q*-axis current ($i_{q1}+i_{q2}$), and is the sum of the power generated by each winding set;
- The *q*-axis current, known as the active current, dominates the power exchange process;
- The input current from the PEMFC can be controlled by regulating the *q*-axis current $i_{q1}$;
- Discharge power of PEMFC can be distributed to the vehicle and battery in different proportions by regulating the *q*-axis current $i_{q2}$.

**Figure 7:**
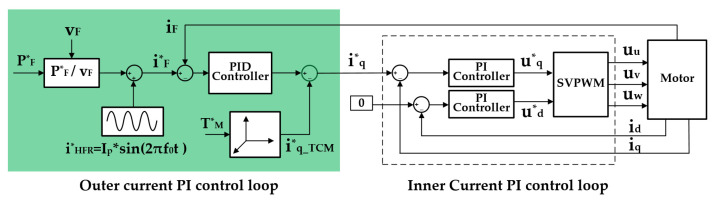
Realization of HFR perturbation current based on the proposed control method.

In view of the above discussion, the input current control (ICC) was introduced to improve the problem of the deterioration of the PEMFC current, as illustrated in Figure 7. The ICC control loop took the discharge current of PEMFC as the control target directly, and yielded the optimal control for the PEMFC current. The reference for HFR perturbation current:

(16) $$i_{HFR\_ref}=I_{p}\sin\left(2\pi ft+\theta \right)$$

is added on the current requirement for PEMFC derived from the power demand. In Equation (16), $I_{p}$ is the amplitude of the HFR perturbation current, typically 1~10% of the current load [24], f is the HFR measurement frequency, and 300 Hz [9] is selected in this paper. The input current from PEMFC is regulated through a PID controller and the obtained output:

(17) $$i_{q1\_PID}=k_{P1,dc}\left(\frac{P_{f\_ref}}{u_{f}}+i_{HFR\_ref}-i_{f}\right)+k_{I1,dc}\int^{\;}\left(\frac{P_{f\_ref}}{u_{f}}+i_{HFR\_ref}-i_{f}\right)dt\;,\;$$

feeds into the *q*1-axis current control loop as a part of the reference. In Equation (17), $P_{f\_ref}$ is the power demand for PEMFC determined by the vehicle dynamics and PEMFC’s optimal current characteristic, $u_{f}$ and $i_{f}$, are the sampled dc voltage and current of PEMFC, $k_{P1,dc}$ and $k_{I1,dc}$ are the proportional and integral coefficients of the PID controllers. In order to improve the control stability, a differential coefficient was selected to zero and a feedforward part was introduced:

(18) $$i_{q1\_TCM}=\frac{2}{3}\frac{T_{1\_ref}}{p_{0}\Psi_{f}}$$

where $T_{1\_ref}$ is the torque demand to winding set 1 derived from the power distribution. The torque–current model expressed in Equation (18) was deduced from Equation (15).

The sum of the output of the PID controller $i_{q1\_PID}$ and feedforward current $i_{q1\_TCM}$ fed into the *q*1-axis current control loop as a total reference:

(19) $$i_{q1\_ref}=i_{q1\_PID}+i_{q1\_TCM}\;,$$

This topology formed a double loop control system that is composed of a low-speed outer loop to control the input current and a high-speed inner loop to control the phase current of the motor. 

The feasibility of the proposed method to a practical system regarding measurement accuracy is an important evaluation topic. The measurement accuracy depends on two main factors, estimation algorithm and signals processing. Both aspects have been studied by some predecessors [9,10] and will not be analyzed in this paper to avoid deviating from the subject. Both the current perturbation and voltage response were weak signals relative to those used for driving the vehicle. To abstract the weak signals, differential signal processing circuits with a high common-mode rejection ratio (CMRR) can be used, and the phase delay and amplitude attenuation should be calibrated and compensated in the estimation algorithm.

### 4.2. Torque Ripple Compensation Based on the Energy Exchange Principles of DWM

**Figure 8:**
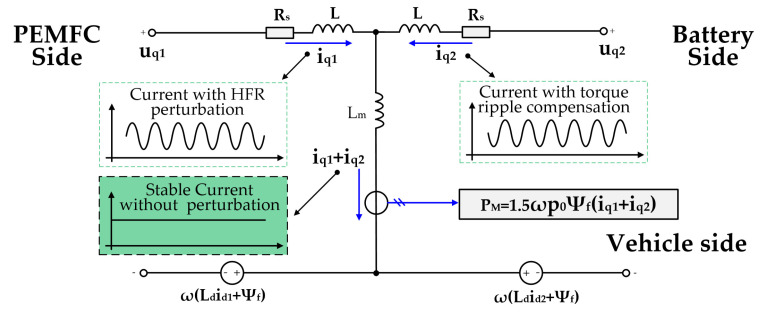
*q*-axis equivalent circuit of the dual-winding motor.

As shown in the *q*-axis equivalent circuit of DWM in Figure 8, the applied HFR perturbation current will cause a fluctuation of the current $i_{q1}$, which, in turn, will cause a fluctuation of the overall current on the *q*-axis:

(20) $$i_{q}=i_{q1}+i_{q2}\;,$$

and then torque ripple occurs. According to Equation (15) and the revealed energy exchange principles, the energy fluctuation caused by current $i_{q1}$ can be transferred to the battery rather than the vehicle, by means of regulating the current $i_{q2}$. In another words, the ripple on current $i_{q1}$ is compensated by current $i_{q2}$, and the fluctuation of the overall *q*-axis current, or torque, is then optimized.

The average value of the current $i_{q1}$ in the past can be estimated using the moving average (MA) method:

(21) $$i_{q1\_avg}\left(t\right)=\beta i_{q1\_avg}\left(t-1\right)+\left(1-\beta \right)i_{q1}\left(t\right)\;,$$

where β is the weighting coefficient, $i_{q1}\left(t\right)$ represents the *q*1-axis current sampled at this cycle, and $i_{q1\_avg}\left(t-1\right)$ represents the estimated average value of the current $i_{q1}$ at the last cycle. Thus, amplitude of the current ripple occurring on the *q*1-axis can be expressed as:

(22) $$i_{q1\_ripple}\left(t\right)=i_{q1}\left(t\right)-i_{q1\_avg}\left(t\right)\;.$$

As mentioned above, the energy fluctuation shall be transferred to the battery by means of regulating the current $i_{q2}$. Therefore, an extra current reference, which is of the same amplitude but opposite phase relative to the estimated signal $i_{q1\_ripple}$, shall be added on the control reference of current $i_{q2}$.

### 4.3. Decoupling Control for the Inner Loop 

**Figure 9:**
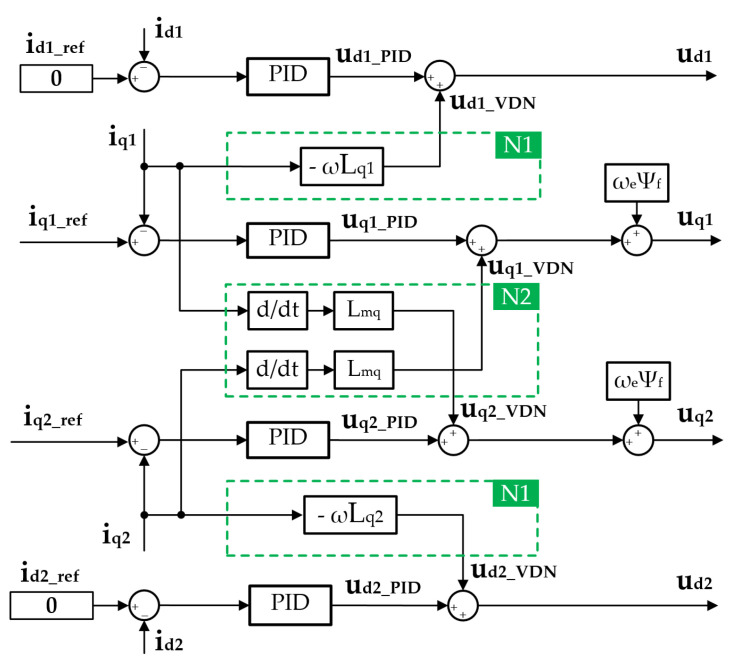
Block diagram of the inner current control loop with decoupling network.

Equations (6)–(9) indicated that there was a significant coupling effect within DWM. The coupling relationship between windings was dominated by mutual inductance $L_{md}$ and $L_{mq}$. The coupling relationship within each winding was dominated by self-inductance $L_{d}$ and $L_{q}$. The current transient in one winding led to voltage disturbance to the other winding, which may deteriorate the dynamic behavior of the inner current control loop when performing an HFR measurement. Under the condition that the actual value of current $i_{d1}$ and $i_{d2}$ are approximately equal to zero, the decoupling voltage equations can be simplified as:

(23) $$u_{d1\_VDN}=-\omega_{e}L_{q1}i_{q1}$$

(24) $$u_{q1\_VDN}=L_{mq}\frac{di_{q2}}{dt}$$

(25) $$u_{d2\_VDN}=-\omega_{e}L_{q2}i_{q2}$$

(26) $$u_{q2\_VDN}=L_{mq}\frac{di_{q1}}{dt}$$

that stand for the cross-coupled voltage on each axis. Further, block diagram of the inner current control loop with decoupling network is depicted in Figure 9. The inner control loop for the motor phase current was based on the PID controllers of current $i_{d1}$, $i_{q1}$, $i_{d2}$, and $i_{q2}.$ The voltage decoupling network N1 for each winding set was introduced to compensate for the coupling effect of the *q*-axis current on the *d*-axis voltage. The voltage decoupling network N2 across two winding sets was used to compensate the coupling effect between the two *q*-axes. The calculated decoupling voltage was added on the regulated voltage produced by the inner PID controllers.

In summary, the voltage decoupling network was equivalent to a feedforward control, which predicts the coupling effect on each winding according to the detected current, and applies compensation voltage to the *d*/*q*-axis in a feedforward way, rather than a feedback way. Thus, the control performance of the motor phase current under the HFR measurement condition can be improved.

## 5. Simulation and Discussion

**Figure 10:**
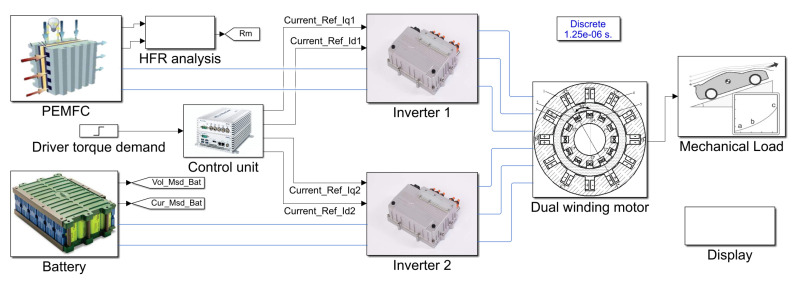
Overview of the system simulation model.

A simulation model of the drive system was established using MATLAB/Simulink R2018a, shown in Figure 10. The PEMFC and DWM models were established based on the mathematical models in Section 3. The inverter physical model was built up based on the electrical elements provided by Simulink library models, and the battery was simplified to a second order equivalent model. The control method, proposed in Section 4, was modeled by Simulink and packaged in the control unit model. The parameters of the DWM and PEMFC, typically used for the fuel cell and traction motor of electric vehicle, are given in Table 2.

**Figure 11:**
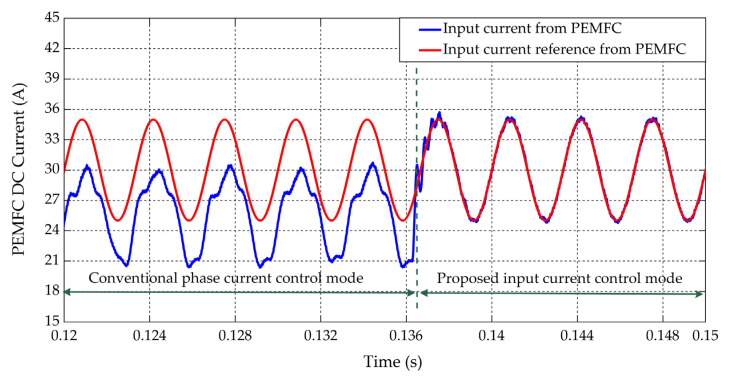
Shaping of the PEMFC current under the conventional phase current control and the proposed input current control.

**Figure 12:**
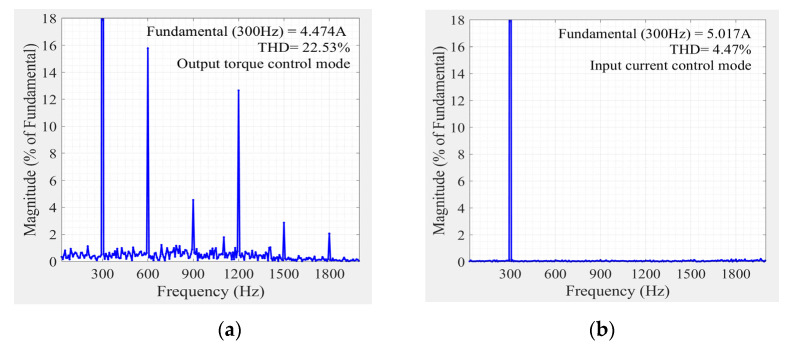
FFT of the PEMFC current: (**a**) Under the conventional phase current control; (**b**) Under the proposed input current control.

Figure 11 shows the partial enlarged signals of the PEMFC current under a given current reference with the HFR perturbation at a typical speed (1500 rpm). In this simulation, the control method was switched from conventional phase current control to the proposed input current control mode for comparison. Results showed that shaping of the current deviates from the reference under the conventional method due to the uncontrollability of the dc current. While under the proposed method, the dc current tracks the reference more accurately. Fast Fourier transform (FFT) results indicated that the total harmonic distortion (THD) of the PEMFC current was reduced from 22.53% to 4.47% of the fundamental, as shown in Figure 12.

**Figure 13:**
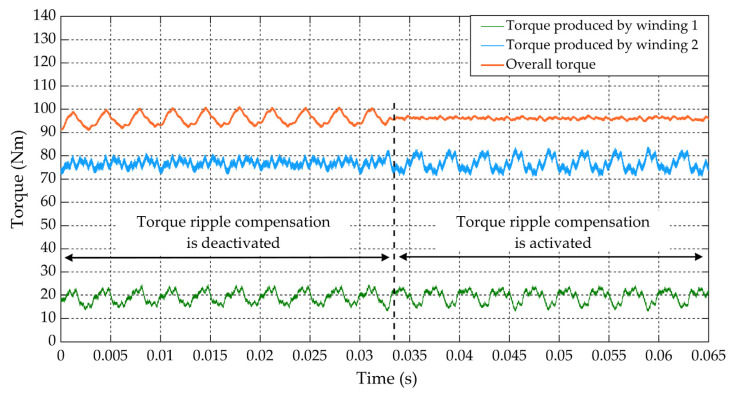
Suppression of the torque ripple under the HFR measurement condition.

Figure 13 shows the comparison of the torque ripple before and after the activation of the torque ripple compensation at 1500 rpm. Without the compensation, the injection of the 300 Hz perturbation current with ±5 A amplitude led to about ±4.5 Nm torque ripple of the same frequency. That is unacceptable for vehicle comfortability. When the torque ripple compensation is activated at 0.033 s, a compensation torque with similar amplitude and the same frequency but opposite phase was generated by winding 2. In this way, the undesired energy due to the torque ripple was mainly absorbed by the battery. So, torque ripple on the mechanical port was suppressed from about ±4.5 Nm to ±0.5 Nm significantly.

To verify the effectiveness of the voltage decoupling network N1 in each winding set, the dynamic response of the PID controller for current $i_{d1}$ was investigated under dynamic conditions of the current $i_{q1}$. Simulation was implemented in three stages, expressed in Table 3.

**Figure 14:**
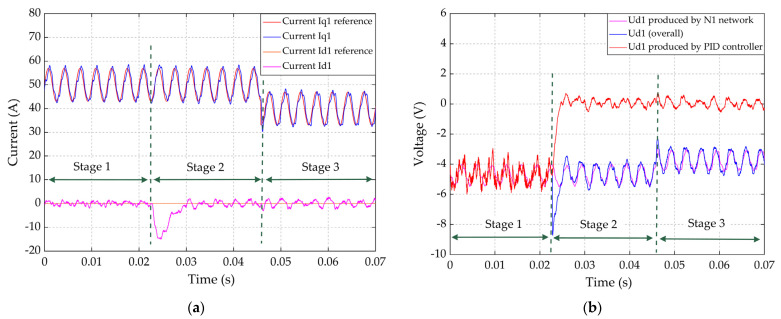
The dynamic response of the current and voltage on *d*/*q*-axis under HFR measurement and transient load change conditions in three stages: (**a**) Reference and sampled current on the *d*1-axis and *q*1-axis, (**b**) *d*-axis voltage produced by PID controller and the N1 network and their sum $u_{d1}$.

Results are shown in Figure 14. Because N1 is deactivated in stage 1, there was no decoupling for compensation. The PID controller provided the total amount of voltage $u_{d1}$, including the cross-coupled voltage ($-\omega_{e}L_{q1}i_{q1}$) induced by current $i_{q1}$, shown in Figure 14b. The high frequency fluctuation of $i_{q1}$ caused by HFR measurement will directly cause the high frequency fluctuation of PID controller output voltage, which will degrade the stability of the inner control loop. In stage 2, N1 was activated and provided the decoupling voltage compensation in a feedforward way, then the PID controller output voltage decreased to around zero and consequently, its high frequency fluctuation significantly reduced, shown in Figure 14b. In stage 3, although a transient load change was imposed on current $i_{q1}$ from 50 A to 40 A, the PID controller output voltage and HFR measurement were not influenced significantly. 

To verify the effectiveness of the decoupling network N2 across two wingding sets, the dynamic response of the PID controller for current $i_{q1}$ was investigated under a disturbance from current $i_{q2}$ resultant from torque ripple compensation. Simulation is implemented in three stages, as expressed in Table 4.

**Figure 15:**
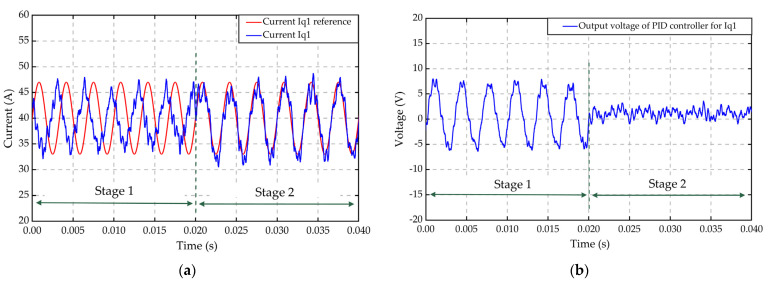
The dynamic response of the current and voltage on the *q*-axis under HFR measurement and torque ripple compensation conditions in two stages: (**a**) Reference and sampled current on *q*1-axis, (**b**) *q*1-axis voltage produced by the PID controller.

Results are shown in Figure 15. N2 was deactivated in stage 1 and the PID controller provided the total regulation voltage $u_{q1}$, including the cross-coupled voltage ($L_{mq}\frac{di_{q2}}{dt}$) induced by current $i_{q2}$. This coupling effect caused not only a fluctuation of the PID controller output shown as Figure 15b, but also a phase shift of current $i_{q1}$ between the reference and actual value shown as Figure 15a. In stage 2, N2 was activated and compensated the cross-coupled voltage disturbance ($L_{mq}\frac{di_{q2}}{dt}$) in a feedforward way. Hence, the fluctuation of the PID controller’s output $u_{q1\_\mathrm{PID}}$ was obviously reduced, and the actual current $i_{q1}$ tracked the amplitude and phase of the reference more accurately.

## 6. Conclusions

This paper studied one of the neglected topics in fuel cells-related literature based on the dual-winding motor drive application. Based on the dual-winding motor drive system architecture studied by predecessors, this paper proposed a new control method for the dual-winding motor to realize high-frequency resistance measurement for fuel cells and normal drive control simultaneously without using a high-cost dc-dc converter. Simulation results verified the effectiveness of this method. Thus, it represents a promising solution of water management for the fuel cell vehicle based on the dual-winding motor drive system in a more economical way. As part of the proposed method, this paper revealed the energy exchange principles among the mechanical port and the two electrical ports of the dual-winding motor in a *d–q* rotational reference frame. This is meaningful for the study of control algorithms and based on that the torque ripple compensation is realized by the regulation of the *q*-axis current.

To be used further in a generic system, more related work needs to be performed to make the proposed method applicable to a dual-winding motor with a generic angular displacement between two winding sets. With such knowledge, an improved dual-winding motor drive technology could be possible, which could ensure the durability and cost necessary for achieving commercial success.

## Figures and Tables

**Table 1:** Operation modes of the drivetrain.

Operation Mode	Sub-Mode	PEMFC	Battery	Motor	Vehicle
Single-source mode	S1	Null	O	I	Traction
	S2	Null	I	O	Braking
Dual-source mode	D1	O	O	I	Boost traction
Energy transfer mode	R1	O	I	I	Charging-traction
	R2	O	I	O	Charging-braking

**Table 2:** Simulation experiment parameters of DWM and PEMFC.

Parameter Description and Its Mathematical Notation	Value
$\mathrm{Phase}\;\mathrm{resistance},\;R_{1},R_{2}$	91.8 mΩ
$\mathrm{Self}-\mathrm{inductance}\;\mathrm{on}\;d-\mathrm{axis}\;\mathrm{and}\;q-\mathrm{axis},\;L_{d1},\;L_{d2},\;L_{q1},\;L_{q2}$	0.5 mH
Mutual inductance on *d*-axis and *q*-axis, *L_md_*, *L_mq_*	0.9 mH
$\mathrm{Flux}\;\mathrm{linkage}\;\mathrm{of}\;\mathrm{the}\;\mathrm{magnet},\;\Psi_{f}$	0.0832 Wb
$\mathrm{Pole}\;\mathrm{pair}\;\mathrm{numbers},\;p_{0}$	4
$\mathrm{Cell}\;\mathrm{Nernst}\;\mathrm{voltage},\;E_{n}$	1.2 V
Cell numbers in serials, N	110
$\mathrm{Cell}\;\mathrm{membrane}\;\mathrm{resistance},\;R_{m}$	0.91 mΩ
$\mathrm{Cell}\;\mathrm{double}-\mathrm{layer}\;\mathrm{capacitor},\;\;R_{f}$	1.82 mΩ
$\mathrm{Cell}\;\mathrm{catalyst}\;\mathrm{layer}\;\mathrm{faradic}\;\mathrm{resistance},\;\;k_{p}$	0.85
Frequency of HFR measurement, f	300 Hz
$\mathrm{The}\;\mathrm{amplitude}\;\mathrm{of}\;\mathrm{HFR}\;\mathrm{perturbation}\;\mathrm{current},\;I_{p}$	5 A

**Table 3:** Parameters and the work mode in three stages.

Parameters and the Work Mode	Stage 1	Stage 2	Stage 3
Time range	0–22 ms	22–46 ms	46–70 ms
$\mathrm{Amplitude}\;\mathrm{of}\;\mathrm{HFR}\;\mathrm{perturbation}\;\mathrm{on}\;i_{q1}$	±4 A	±4 A	±4 A
$\mathrm{Frequency}\;\mathrm{of}\;\mathrm{HFR}\;\mathrm{perturbation}\;\mathrm{on}\;i_{q1}$	300 Hz	300 Hz	300 Hz
$\mathrm{Load}\;\mathrm{driving}\;\mathrm{current}\;\mathrm{on}\;i_{q1}$	50 A	50 A	40 A
Voltage decoupling network N1	Not activated	Activated	Activated

**Table 4:** Parameters and the work mode in two stages.

Parameters and the Work Mode	Stage 1	Stage 2
Time range	0–20 ms	20–40 ms
$\mathrm{Amplitude}\;\mathrm{of}\;\mathrm{HFR}\;\mathrm{perturbation}\;\mathrm{on}\;i_{q1}$	±4 A	±4 A
$\mathrm{Frequency}\;\mathrm{of}\;\mathrm{HFR}\;\mathrm{perturbation}\;\mathrm{on}\;i_{q1}$	300 Hz	300 Hz
$\mathrm{Torque}\;\mathrm{ripple}\;\mathrm{compensation}\;\mathrm{current}\;\mathrm{on}\;i_{q2}$	Activated	Activated
Voltage decoupling network N1	Not activated	Activated

## Data Availability

Not applicable.
